# Cenozoic global cooling and increased seawater Mg/Ca via reduced reverse weathering

**DOI:** 10.1038/s41467-017-00853-5

**Published:** 2017-10-10

**Authors:** Ann G. Dunlea, Richard W. Murray, Danielle P. Santiago Ramos, John A. Higgins

**Affiliations:** 1 Department of Geology and Geophysics, Woods Hole Oceanographic Institution, 266 Woods Hole Road, Woods Hole, MA 02543 USA; 2 Department of Earth and Environment, Boston University, 685 Commonwealth Ave., Boston, MA 02215 USA; 3 Department of Geosciences, Princeton University, Guyot Hall, Princeton, NJ 08544 USA

## Abstract

Authigenic clay minerals formed on or in the seafloor occur in every type of marine sediment. They are recognized to be a major sink of many elements in the ocean but are difficult to study directly due to dilution by detrital clay minerals. The extremely low dust fluxes and marine sedimentation rates in the South Pacific Gyre (SPG) provide a unique opportunity to examine relatively undiluted authigenic clay. Here, using Mg isotopes and element concentrations combined with multivariate statistical modeling, we fingerprint and quantify the abundance of authigenic clay within SPG sediment. Key reactants include volcanic ash (source of reactive aluminium) and reactive biogenic silica on or shallowly buried within the seafloor. Our results, together with previous studies, suggest that global reorganizations of biogenic silica burial over the Cenozoic reduced marine authigenic clay formation, contributing to the rise in seawater Mg/Ca and decline in atmospheric CO_2_ over the past 50 million years.

## Abstract (web summary)

Reverse weathering reactions on or in the seafloor are a major sink of many elements and alkalinity in seawater. Here, the authors show how reduced rates of reverse weathering may be responsible for global cooling and increased seawater Mg/Ca over the past 50 million years.

## Introduction

The formation of marine authigenic clays is one of the most enigmatic and least understood of the major processes involved in the global geochemical cycling of elements and alkalinity in the ocean^1–4^. Early work on chemical mass balance in the oceans hypothesized that authigenic clay formation via a reverse weathering reaction was one of the principle sinks of Si, Mg, K, Na, and alkalinity from seawater^5, 6^.

1 $${\rm{Si}}{{\rm{O}}_{{\rm{2(s)}}}} + {\rm{Al}}{\left( {{\rm{O}}{{\rm{H}}_{\rm{3}}}} \right)_{{\rm{(s)}}}} + \left( {{\rm{M}}{{\rm{g}}^{{\rm{2 + }}}},{{\rm{K}}^{\rm{ + }}},{\rm{N}}{{\rm{a}}^{\rm{ + }}}} \right) + {\rm{HCO}}_{3({\rm{aq}})}^ - \\ \to {\rm{new}}\,{\rm{clay}}\,{\rm{mineral + C}}{{\rm{O}}}_{2({\rm{aq}})} + {{\rm{H}}_{2}}{\rm{O}}$$

Although the discoveries of high and low-temperature hydrothermal systems at mid-ocean ridges identified a new sink for elements in the ocean^7, 8^, more recent studies indicate that the formation of authigenic clays in both shallow and deep-sea sediments of diverse lithologies is indeed widespread and likely quantitatively important for the geochemical budgets of cations and alkalinity in seawater^9–18^.

In this study, we fingerprint and quantify the abundance of authigenic clays within marine sediment from the largest oceanic province in the world, the South Pacific Gyre (SPG). Drilled during Integrated Ocean Drilling Program (IODP) Expedition 329, the pelagic sediment we examine is completely oxic, homogenous, red−brown, zeolitic, and metalliferous, and also includes abundant authigenic clay minerals^19, 20^. Far from land and in a region of low oceanic primary productivity, sedimentation rates in the SPG are among the slowest in the world ( ~ 0.1–1 m/Myr or μm/year)^19, 21^. Thus, the authigenic clays within the sediment are relatively undiluted by dust and biogenic deposition^22^, making the SPG an optimal location to study the geochemical signatures of and processes driving marine authigenic clay formation.

Our results support previous observations of the components and conditions required to form marine authigenic clays^9–18^ and highlight the importance of volcanic ash and biogenic Si as key reactants in the deep sea. In most ocean regions, the formation of authigenic clay is limited by the presence of reactive silica and we use our results to examine how the geographic reorganization of Si accumulation on the seafloor changed the global production of authigenic clay through time. We propose that shifts in biogenic silica burial decreased authigenic clay formation and could be responsible, in large part, for the increase in seawater Mg/Ca ratio observed over the Cenozoic. Considering the involvement and associated feedback loops of the global carbon cycle, reduced reverse weathering may also have contributed significantly to the decline in atmospheric CO_2_ and thus global cooling over the past 50 million years.

## Results

### The geochemical fingerprint of marine authigenic clay

**Fig. 1:**
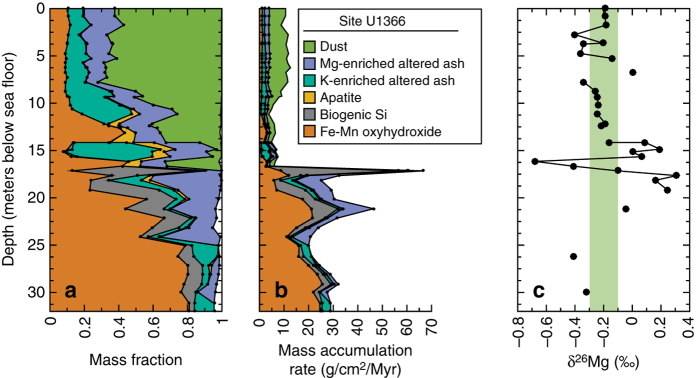
End-member model and δ^26^Mg of bulk sediment at Site U1366. **a** Mass fractions of the six end-members that comprise the deep-sea pelagic sediment at Site U1366, as modeled with constrained least squares multiple linear regression. **b** Mass accumulation rates of each end-member calculated from the modeled mass fractions and total mass accumulation rates^21^. **c** Measured δ^26^Mg (‰) values for the same bulk sediment samples. *Green vertical* shading highlights the samples with δ^26^Mg values within the range of average upper continental composition (0−14 mbsf) and helps identify the samples with heavier δ^26^Mg (14−20 mbsf, to the right of the *green vertical* shading) caused by the incorporation of seawater Mg into authigenic phases

We identify authigenic clay, which is broadly aluminosilicate in composition, in sediment from the SPG using two independent approaches. First, we use geochemical partitioning techniques and construct multivariate statistical mixing models based on a data set including major and trace element concentrations in 138 bulk pelagic sediment samples from IODP Sites U1365, U1366, U1367, U1369, U1370, and U1371^22^. We used Q-mode factor analysis of the data set to identify the authigenic end-members and constrained least square multiple linear regression models to quantify their abundance (i.e., mass fraction) within the bulk sediment (Supplementary Note 1)^22–24^. Including nine major elements (Si, Al, Ti, Fe, Mn, Ca, Mg, K, and P) and a few trace elements (Cr, Rb, Cs), both statistical techniques quantitatively identify that the sediments are a mixture of six end-members, namely, dust, Fe/Mn-oxyhydroxides, biogenic Si, apatite, and two compositionally different altered volcanic ashes (Fig. 1a, b, and Supplementary Note 1, Supplementary Figs. 1−3, Supplementary Table 1). The two altered ashes were sampled from discrete ash layers at Sites U1366 and U1370 and their compositions are enriched in Mg and K, respectively.

**Fig. 2:**
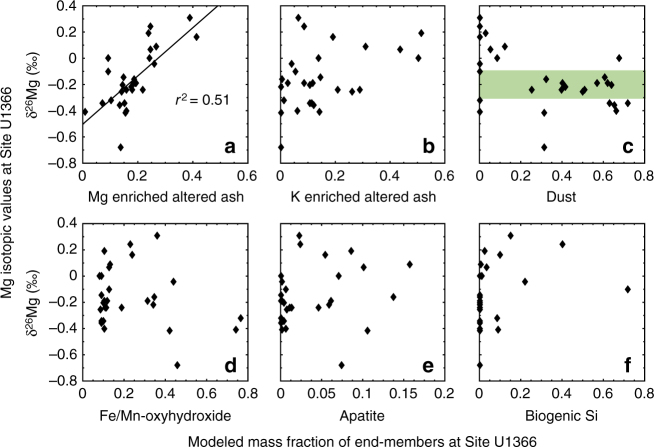
Comparison of two techniques that identify authigenic clay at Site U1366. Measured δ^26^Mg (‰, all *y*-axes) values are plotted against the modeled mass fraction (*x*-axes) for each of the six components of the bulk sediment (**a**–**f**). **a** The significant correlation (*r* ^2^ = 0.51; *P* < 0.01) between δ^26^Mg and the mass fraction of Mg-enriched altered ash is consistent with heavier Mg isotopes being incorporated from seawater into an authigenic clay component. **c** Samples with a high mass fraction of dust plot near the δ^26^Mg values typical of bulk-silicate earth (*green shaded region*), further indicating consistency between the two techniques

Second, we measure the magnesium isotope ratio (δ^26^Mg) of the bulk sediment at Site U1366. Previous studies of both terrestrial^25^ and marine^2^ environments indicate that the incorporation of Mg into authigenic clays involves the preferential uptake of the heavier Mg isotope (^26^Mg) and that the Mg isotopic fractionation associated with clay formation ranges from 0 to + 1.25 per mil (‰) relative to the solution. As a result, authigenic marine clays should be enriched in ^26^Mg compared to seawater (δ^26^Mg = − 0.82‰) and bulk-silicate earth (δ^26^Mg = − 0.29‰). Measured δ^26^Mg values of the Site U1366 bulk sediment ranges from − 0.68‰ to + 0.31‰ (Fig. 1c, Supplementary Table 1). The Mg-enriched altered ash layer used as an end-member in the bulk chemistry statistical models had an even higher δ^26^Mg signature of + 0.52‰, which is one of the highest δ^26^Mg values ever recorded in solid-phase marine sediment and is 1.3‰ heavier than seawater (Supplementary Fig. 4). Other than the ash layer, the bulk sediments that yielded higher modeled mass fractions of the Mg-enriched altered ash in the statistical models had consistently heavier δ^26^Mg values (Fig. 2). Agreement between the two geochemical techniques is strong evidence that the Mg-enriched altered ash represents a clay component that has incorporated Mg directly from seawater into its mineral structure.

### The global marine authigenic clay sink

Quantifying the global sink of seawater elements in authigenic marine clays requires an understanding of both the kinetics and the ingredients that promote authigenic clay formation. Two lines of evidence suggest that the formation of authigenic clays in the SPG was geologically rapid and occurred at or near the seafloor, rather than deeply buried. First, porewater profiles of Mg concentrations and δ^26^Mg are constant with depth at each site^19^ (Supplementary Fig. 5), indicating that clays are not actively removing or releasing Mg into deeply buried porewaters. Second, the linear relationship between the modeled mass fraction of Mg-enriched altered ash and their δ^26^Mg values (Fig. 2) indicates mixing of two end-members with distinct δ^26^Mg compositions. If clay authigenesis had occurred deeper in the sediment column, Rayleigh-type distillation of Mg in the pore-fluid would have resulted in variable δ^26^Mg values for the authigenic clay end-member^2, 26^.

The often-described reverse weathering reaction that forms authigenic clay^5, 9^ requires three ingredients including a reactive Al source (“amorphous aluminosilicate”), a reactive Si source, and cations (e.g., Mg^2+^, K^+^, Fe^2+,3+^, Na^+^). Our isotopic and provenance results agree with this recipe for authigenic marine clays, although differences exist in the source of each reactant supplying the open ocean versus regions closer to continents, as discussed here.

The geochemical provenance models in our study suggest that volcanic ash is the primary precursor substrate to the authigenic clays forming in the deep-sea pelagic sediment in the SPG^22^. Volcanic ash is an excellent candidate to undergo alterations on or within the seafloor because it is deposited as very fine-grained amorphous glass susceptible to dissolution and is known to alter into smectites and zeolites, which have high cation exchange capacities^27, 28^. Studies of porewater profiles and authigenic minerals have hypothesized that altered volcanic ash is a solid phase in marine sediment consuming Mg, K, Li, and other cations from porewaters^13, 27–30^. Volcanic ash is also ubiquitous in bulk marine sediment^31^ in globally significant abundances^32^, and is deposited as discrete layers and/or dispersed ash that has been mixed into bulk sediment from ash layers via bioturbation or the settling of airborne or subaqueous ash. In short, the abundance of volcanic ash in the deep sea is vastly greater than is accounted for in the visible discrete volcanic ash layers. Volcanic ash may also be present in shallower water sediment systems, but eolian dust or riverine sediment may be a more important source of degraded minerals closer to the continents.

At Site U1366, our model results indicate that the abundance of Mg-enriched altered ash is related to the accumulation rate of biogenic Si (Fig. 1). The accumulation of biogenic Si is an important source of reactive Si for authigenic clay formation, in addition to any Si that is also released during ash alteration^14, 28^. Closer to river deltas and continental shelves, dissolved reactive Si may be supplied directly by river or from biogenic siliceous deposition^10, 33^.

Seawater provides an ample supply of Mg^2+^ (and other cations such as K^+^ and Na^+^) to be consumed during reverse weathering reactions. There may also be local sources of Mg within the sediment from the dissolution of aluminosilicate minerals, but these are likely to be relatively minor in early authigenesis compared to contributions from seawater.

In summary, an intimate mixture of biogenic Si with abundant and ubiquitous volcanic ash on or shallowly buried beneath the seafloor appears to be the most conducive environment to forming authigenic clay in deep-sea pelagic sediment. With this recipe we proceed to estimate the rate and extent of authigenic clay formation and quantify its global importance as a sink for seawater Mg and alkalinity and how it may have changed over the Cenozoic.

### Global reorganization of Si burial limits clay formation

In most ocean regions, the availability of reactive Si limits the formation of authigenic clays^9, 15^. The exceptions are in the Southern Ocean, and a few other smaller regions, where rapid accumulation of siliceous material significantly increases the Si concentrations of the porewater in the shallowly buried seafloor and the supply of Al limits the formation of authigenic clays^16^. Thus, changes in the geographic distribution, accumulation, and mixing of these components control the extent to which authigenic clays form in sediments globally.

Reconstructions of the abundance and spatial patterns of silica deposition over the Cenozoic have revealed profound changes in the distribution and mode of silica burial^34–37^. At present, the accumulation of siliceous material is primarily focused in the Southern Ocean. During the Paleogene, and in particular during the Early Eocene Climatic Optimum (EECO), biogenic silica accumulation and its diagenetic products (e.g., porcellanites and chert)^38^ were more widely deposited throughout the oceans^34–37^. The global occurrence of chert reached a peak, despite overall open ocean biosiliceous productivity being relatively low^36^. The presence of metals within opal and siliceous diagenetic products is known to lower mineral solubility, and authigenic clay minerals are commonly found in intimate association^16, 17, 39^. Together these observations suggest a global silica cycle in the Paleogene that was considerably more favorable for the formation of authigenic marine clays.

In the later Eocene and Oligocene, biogenic Si accumulation shifted from being dispersed in the open ocean to being focused beneath more localized upwelling regimes, reducing the spatial extent of reactive Si available to the seafloor. This change is documented most notably in the Southern Ocean where formerly silica-poor sediments were replaced by the largest sink of biogenic silica on Earth^37^. The trigger for the shift may have been changes in ocean circulation from the opening or closing of ocean gateways^40^, and/or the concurrent evolutionary radiation and proliferation of diatoms, which require concentrated silicic acid to grow^37^. Regardless of the trigger, a large spatial shift in the accumulation of siliceous material through the Cenozoic is clearly evident in the sedimentary record.

A consequence of the redistribution of global biogenic silica burial over the Cenozoic is that two of the critical ingredients to form marine authigenic clays—reactive Al and reactive Si—became geographically isolated from one another. Given that marine authigenic clay formation appears to be limited by the availability of one or both of these ingredients^9, 16^, the un-mixing of these components also likely resulted in a decline in the global marine authigenic clay sink over the Cenozoic. A reduction in authigenic clay would decrease the Mg removed from seawater and may explain most of the change in global Mg budgets observed over the Cenozoic.

### Reduced authigenic clay formation increased seawater Mg/Ca

Reconstructions of seawater Mg/Ca from a wide range of geochemical proxies over the Cenozoic indicate that seawater Mg/Ca has undergone a 2 to 3-fold increase, driven by both an increase in seawater Mg and a decline in seawater Ca^41, 42^. Higgins and Schrag^43^ used reconstructions of the δ^26^Mg composition of seawater from bulk foraminifera and fossil corals over the Cenozoic to show that the increase in seawater Mg flux was  ~ 3 Tmol/year and likely related to changes in the aluminosilicate flux, rather than variations in carbonate deposition or carbonate weathering. That is, the  ~ 3 Tmol/year change was caused by an increase in Mg sources from silicate weathering or a decline in Mg sinks in authigenic clays forming in marine sediments or low-temperature hydrothermal systems.

**Fig. 3:**
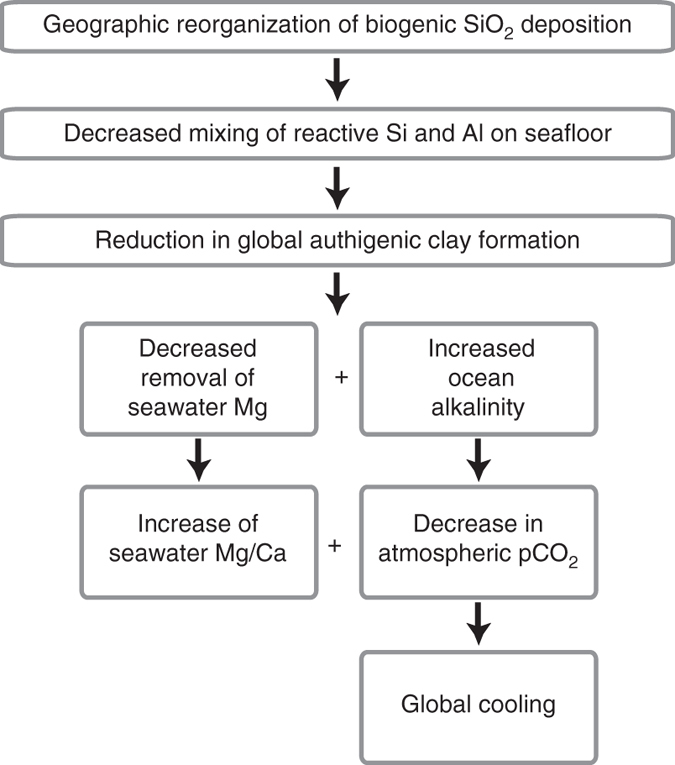
Mechanism for reverse weathering affecting seawater Mg/Ca and climate. The proposed mechanism by which geographic reorganization of biogenic Si deposition and a reduction of global authigenic clay formation could cause an increase in seawater Mg/Ca and global cooling, as is observed over Cenozoic. The decreased sink of alkalinity in the ocean would be balanced by a reduction in global silicate weathering until the charge-weighted masses of sources are balanced by the sinks of alkalinity

The mechanism favored by Higgins and Schrag^43^ was a decline in the Mg sink in marine clays due to cooler global temperatures slowing reaction kinetics. They speculated that the sink of Mg in authigenic clays might have a temperature dependence similar to off-axis low-temperature hydrothermal systems (i.e., basalt alterations from fluid flowing through the basement rock), but there are significant uncertainties in this comparison^44–49^. Here, we propose an alternative explanation that is independent of, but may complement, any temperature-dependent Mg removal in either system. The shift in the spatial distribution of biogenic silica accumulation in the ocean over the Cenozoic and an un-mixing of the ingredients required for the formation of marine authigenic clays caused a decline in the Mg sink in sedimentary authigenic marine clays (Fig. 3)

.

Our study provides strong empirical evidence that Mg in the SPG clays was taken up from seawater and allows us to produce first-order, quantitative constraints on the importance of authigenic clay formation as a sink for Mg. Given the enrichment of Mg in the altered ash component from this study, we extrapolate our results to estimate that typical deep-sea sediment removes ~ 0.02 Tmol of Mg from seawater per year (Supplementary Note 2). Although this present-day Mg sink in these pelagic environments is small, Si-enriched bulk sediment sampled from near the chert layer at Site U1365 indicate higher rates of Mg removal that would constitute a significant sink of seawater Mg if occurring on a global scale. For example, if covering 50−100% of the seafloor, this type of sedimentation would remove 0.4−0.8 Tmol of Mg from seawater per year (Supplementary Note 2). These estimates are  ~ 20−35% of the modern riverine influx of Mg from silicate weathering and are the same order of magnitude ( ~ Tmol) as the changes required by models to drive the observed increase in seawater Mg/Ca over the Cenozoic^43^.

The amount of Mg removed by authigenic clay formation is almost certainly even higher in more rapidly accumulating sediments such as river deltas, continental shelves and margins. For example, in the Amazon Delta and mobile mud belts that extend ~ 1600 kilometers away from the continent, ~ 90% of the Si deposition is transformed into authigenic marine clays during burial^10, 33, 39^. Because Si limits authigenic clay formation in these environments, enhanced deposition of biogenic Si during the Paleogene would have promoted the formation of additional authigenic marine clays. In summary, our calculations, combined with estimates for clay formation in shallow water environments^10^, suggest that marine authigenic clays remove Mg on a scale that could explain the change in seawater Mg/Ca over the Cenozoic.

### Cenozoic global cooling via decreased reverse weathering

The apparent decline in atmospheric CO_2_ and associated global cooling through the Cenozoic is commonly attributed to various mechanisms including an increase in the rate of global silicate weathering^50, 51^, changes in weatherability^52, 53^, a decline in volcanic outgassing^54, 55^, or some combination of the three. Here, we propose a fourth possibility—a decrease in reverse weathering in marine sediments. The mechanism is possible because reverse weathering is predicted to remove alkalinity from seawater via the reaction described in Eqn (1) with the total magnitude of the alkalinity sink depending on the relative importance of the reactant cations in the authigenic clay (weighted by charge).

A reduction in the amount of authigenic clay formation would reduce the alkalinity consumed by reverse weathering leading to an increase in the ocean’s alkalinity inventory (sources > sinks), an increase in seawater pH, and a decline in atmospheric CO_2_ (Fig. 3). The decrease in atmospheric CO_2_ would, in turn, result in global cooling and lowered rates of continental silicate and/or seafloor weathering until the point at which it balances the reduced alkalinity sink in authigenic marine clays (sources ≈ sinks). This mechanism is capable of changing rates of continental silicate weathering without changing either the rate of volcanic outgassing^54, 55^ or the rate constant for continental weatherability^52, 53^ and as a result represents a unique way of modulating the global carbon and alkalinity cycles and Earth’s climate on geologic timescales.

Assuming the  ~ 3 Tmol/year change in Mg sink over the Cenozoic predicted from models of Mg/Ca ratios and δ^26^Mg composition of seawater is driven entirely by changes in reverse weathering, the corresponding change in alkalinity consumption (CO_2_ production) is 6 × 10^12^ alkalinity equivalents per year (or 3 Tmol C/year). That amount is ~ 25% of the present-day CO_2_ consumption from silicate weathering ( ~ 12 Tmol C/year)^56^ and is roughly similar to the budgets of Mackenzie and Garrels^5^ which estimated that reverse weathering reactions involving Mg removed  ~ 30% of the river influx of alkalinity. Additional reverse weathering reactions involving Na^+^ and K^+^ may increase the importance of this alkalinity sink. Even when considering only reverse weathering reactions associated with Mg (that is, neglecting the potential contributions of Na and K alterations), our first-order estimates indicate that this change would result in a doubling of atmospheric CO_2_ from common era (280 p.p.m. to 590 p.p.m.), assuming a global carbon cycle at steady-state and silicate weathering that depends on pCO_2_ (Supplementary Note 3). This estimate is on the low end but within the range of atmospheric CO_2_ levels in the mid to late Eocene^57^, suggesting that a decline in the marine authigenic clay sink may have played an underappreciated and significant role in facilitating the decline in atmospheric CO_2_ and associated global cooling over the Cenozoic.

## Methods

### Element concentrations

The data set of bulk sediment major, trace, and rare earth element concentrations used in this study is from Dunlea et al.^21, 22^, in which are described the analytical methods and age model in detail. The same acid digestions of samples that were prepared and analyzed for their trace and rare earth element compositions were also processed and analyzed for Mg isotopes at Princeton University, using techniques described below.

### Multivariate statistical modeling

The Q-mode factor analysis and constrained least squares multiple linear regressions were performed using MATLAB codes published in Pisias et al.^23^, and Dunlea and Murray^24^. Many iterations of the QFA and CLS analyses were run with different sample selections and element menus to check the robustness and stability of the results. Additional details of the statistical modeling and stability tests are discussed in Supplementary Note 1.

### Magnesium isotopes

Mg ions were separated with an automated ion chromatography (IC) Dionex ICS-5000 + IC system coupled with a Dionex AS-AP fraction collector. Samples were then injected with 0.2% HNO_3_ and passed through a Dionex CS-16 cation-exchange column using a methyl-sulfonic acid (MSA) eluent at a flow rate of 1 ml/min. The conductivity of each sample was measured and the sample was collected during the desired time window. Including column washing, elution time per sample ranges from 30 to 50 min, depending on the sample matrix. Blank levels yielded < 5 ng for Mg, or < 1% of the sample mass (500 ng for Mg). Following ion separation, samples were treated with concentrated HNO_3_, dried and re-diluted to 150–200 p.p.b. in 2% HNO_3_, and analyzed on the Thermo Scientific Neptune Plus multi-collector inductively coupled plasma mass spectrometer (MC-ICP-MS) at Princeton University for their Mg isotope composition.

Sample-standard-sample bracketing was used to correct for instrumental mass fractionation^58^ and measurements of inter-laboratory standards confirm the accuracy of these analytical methods. Mg isotopic compositions are reported using delta notation by which sample Mg isotope ratios are calculated relative to the standard DSM-3. The δ^26^Mg of the Cambridge-1 standard measured at  − 2.59 ± 0.06‰ and agrees well with measurements of other laboratories^59^.

### Data availability

The element concentration data used in this study are included in five tables in the supporting information files of Dunlea et al.^22^ and can be also downloaded from the EarthChem Library (http://get.iedadata.org/doi/100603, doi:10.1594/IEDA/100603)^60^. The δ^26^Mg values are reported in Supplementary Table 1 of this study. Any additional data may be obtained from AGD.

## Electronic supplementary material

Supplementary Information
