# Supplementary material for: Cenozoic global cooling and increased seawater Mg/Ca via reduced reverse weathering
Source: Nat Commun. 2017 Oct 10;8:844. doi: 10.1038/s41467-017-00853-5 (PMC5635029; doi:10.1038/s41467-017-00853-5)
Supplement: Supplementary file 1 — Supplementary Information [file 41467_2017_853_MOESM1_ESM.pdf]

Supplementary Note 1. Multivariate Statistical Modeling: General approach and stability of results

Q-mode Factor Analysis (QFA)

Results from the QFA described by *Dunlea et al.* (2015a)[1] indicate that four factors explain 96% of the variance of a 22-element menu of 138 clay-dominated pelagic sediments from Sites U1365, U1366, U1367, U1369, U1370, and U1371. The factors were interpreted as an aluminosilicate component (i.e. dust), Fe/Mn-oxyhydroxide, carbon-fluoroapatite enriched in REEs or other elements concentrated during periods of slow sedimentation rates, and biogenic Si. *Dunlea et al.* (2015a, their page 7)[1] specifically noted that the remaining 4% of the variability could be explained by additional factors that were statistically insignificant for their study, but that could provide important clues to the formation of authigenic phases. Two of these additional factors had high factor scores for Mg and K, respectively, which the authors interpreted as likely representing Mg-enriched and K-enriched altered ashes and/or authigenic minerals (*Dunlea et al*., 2015a, their Section 5.2)[1]. Therefore, for this current study we focus our statistics on these authigenic clay end-members in the QFA analyses.

To specifically target authigenic clay, we performed a 12-element QFA on the 138 pelagic sediment samples from the same IODP sites as *Dunlea et al.* (2015a)[1]. The element menu consisted of 9 major elements (Si, Al, Ti, Fe, Mn, Ca, Mg, K, and P) and three trace elements (Cr, Rb, Cs), all of which have been shown to be elements among the least affected by authigenic processes and effective in distinguishing the different dust and ash sources[1].

In this analysis, six factors explain 99% of the variability of the dataset. Factors 1-4 directly correspond to the factors defined in *Dunlea et al.* (2015a)[1] and we interpret them as an aluminosilicate dust component, Fe/Mn-oxyhydroxide, apatite, and biogenic Si, which explain 62%, 17%, 11%, and 4% of the variability within the dataset, respectively. The fifth factor explains 3% of the variability, has high factor scores for Mg and corresponds to the Mg-enriched ash factor of *Dunlea et al.* (2015a)[1]. The K-enriched factor of *Dunlea et al.* (2015a)[1] is represented in the sixth factor in this current study, and explains 2% of the variability with high factor scores for Al, Ti, K, Cr, and Cs.

To check the robustness of the factors, many iterations of the QFA were performed on a variety of appropriate element menus, each of which included a statistically legitimate number of elements (< 17 for 138 samples, as defined by *Reimann et al.* (2002)[2]) and varying sample size and sample selection. The six factors are consistently reproduced, which gives confidence in the strength of the statistical outcome. The six factors also agree with the mixing trends observed in element x vs. y plots and ternary diagrams (Supplementary Fig. 1 and 2).

If we force an iteration of the QFA to explain the dataset variability with seven factors, the seventh factor has high factor scores for Al, Ti, Rb, and Cs, and expresses a factor score pattern similar to the mafic basalt end-member defined in the aluminosilicate QFA of *Dunlea et al.* (2015a)[1]. However, because it explains only < 1% of the variability, we consider it statistically insignificant and ignore it in this study.

Constrained Least Squares Multiple Linear Regression Modeling

Following the principles of *Pisias et al.* (2013)[3] and using the abovementioned QFA results as guidance, we constructed a constrained least square (CLS) multiple linear regression model of the SPG bulk pelagic sediment dataset to quantify the proportions of each end-member that mixed to create each sample. We fit a CLS model to the 12-element, 138 pelagic clay sample dataset using six end-member compositions that correspond to the six QFA factors. We tested thousands of different end-member composition combinations using the technique of *Dunlea and Murray* (2015)[4]. The resultant model that produced the best correlation coefficients and explained the most data included Post-Archean average Australian shale (i.e., PAAS)[5], metalliferous sediment[6], fish debris[7], pure elemental Si, a K-enriched altered ash[1], and an Mg-enriched ash[1]. The coefficients of determination are as follows: Si (1.00), Al (0.98), Ti (0.69), Fe (0.99), Mn (0.93), Ca (0.89), Mg (0.47), K (0.95), P (0.89), Cr (0.36), Rb (0.88), and Cs (0.59). Even considering the variability in these coefficients, they are all statistically significant due to the very large size of the data set. See Supplementary Fig. 3 for results of CLS model at Sites U1365, U1366, U1369, and U1370.

Supplementary Note 2. Calculating the magnitude of Mg removed by deep-sea authigenic clays

The concentration of authigenic clays in slowly accumulating pelagic sediment may be relatively high, but are they important as a sink for Mg and alkalinity in the global ocean? Models of the global seawater Mg budget estimate the removal of Mg from seawater to be 2.75 to 4.1 Tmol/yr, with low-temperature hydrothermal/clay reactions

accounting for 0.6 to 1.5 Tmol/yr of the total Mg uptake[8]. As is reported in the main text, our calculations suggest that only 0.02 Tmol/yr are taken up into global pelagic sediment. Assuming steady state, this is based on parameters typical of the SPG pelagic clays, including a dry bulk density of 0.35 g of bulk sediment per cubic centimeter[39], a bulk sediment that is comprised of ~20% of an ash enriched in Mg by 3.9 wt.% beyond the expected value for the precursor unaltered ash[1], an average sedimentation rate of 1 m/Myr [10], and the global spatial area of authigenic clay-bearing pelagic sediment of 1.8 x 10^8 km^2 (~50% of the total seafloor)[11]. Thus, deep-sea pelagic sediment, the ocean's most spatially extensive sedimentary lithology, currently is not a significant sink of Mg in the modern ocean.

For the calculation of Mg uptake into more Si-rich sediment, we considered the characteristics of the Si-enriched bulk sediment sample modeled in this study closest to the chert layer at Site U1365 (at 73.9 mbsf). The high fraction of Mg-enriched altered ash and relatively fast accumulation rates indicate more Mg being taken up into clays. Assuming this sample approximates Si-rich deposits from the early Cenozoic[(e.g.,12, 13)], we changed the parameters in the previous calculation to be similar to this Si-enriched sample. The calculation used a dry bulk density of 0.4 g of bulk sediment per cubic centimeter[9], 66% of the sediment being Mg-enriched altered ash, and a sedimentation rate of 5 m/Myr. With this type of sedimentation occurring across 50% to 100% of the seafloor, 0.4 to 0.8 Tmol of Mg would be removed from seawater every year. This is 25-33% of the modern riverine influx of Mg from silicate weathering and is the same order of magnitude (~Tmol) as that required to drive the observed increase in seawater Mg/Ca over the Cenozoic[8].

Supplementary Note 3. Calculating changes in atmospheric CO_2 caused by changes in reverse weathering

Our results suggest that the Early Eocene had an additional 6 x10^{12} alkalinity equivalents/yr (alk eq/yr) from silicate weathering. Exactly how much higher atmospheric CO_2 and temperatures needed to be in the Early Eocene to increase chemical weathering by that amount depends on a large number of factors. Here, we perform a first-order estimate by assuming a global carbon cycle at steady state and silicate weathering that depends on pCO_2 according to:

$$F_{SW}(t) = F_{SW}(t_m) \times \left({pCO_2(t)} \big/ {pCO_2(t_m)} \right)^g \qquad (2)$$

where $F_{SW}(t_m)$ and $pCO_2(t_m)$ are the silicate weathering flux and atmospheric CO_2 in the modern era, 24 x 10^{12} alk eq/yr and 280 ppm, respectively. g is a constant that represents the complex set of processes that link atmospheric CO_2 and global silicate weathering rates (i.e., temperature, runoff, 'weatherability', etc)[14-17] and is set at 0.3, consistent with previous studies[18]. We estimate $F_{SW}(t)$, the rate of silicate weathering in the past, to be 30 x 10^{12} alk eq./yr. That is, 6 x 10^{12} alk eq/yr higher than the modern era to balance the higher rates of reverse weathering. Solving for $pCO_2(t)$ shows the change in reverse weathering results in an approximate doubling of atmospheric CO_2 from 280 ppm as seen in the modern era to 589 ppm.

Supplementary Figure 1. X vs. Y element plots. Element concentrations of bulk sediment samples from seven sites (colored shapes) in the South Pacific Gyre[1] and reference compositions of average rocks, ash layers, and end-members[1, 5, 6, 19-22] (black shapes) are plotted on x vs. y diagrams. The trends in the bulk sediment samples support the interpretations of the multivariate statistics and suggest these element concentrations can be explained by a mixing of Mg-ash, K-ash, and Post-Archean average Australian Shale (PAAS) compositions.

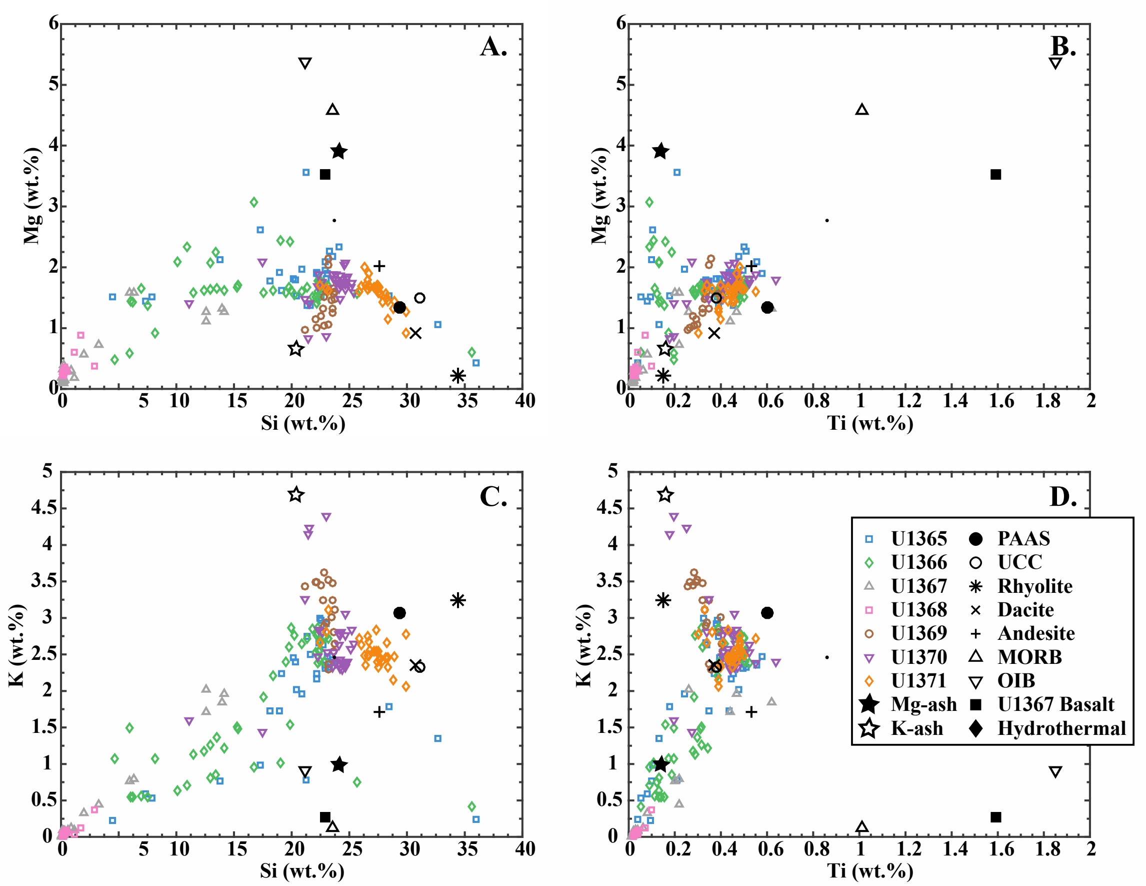

Supplementary Figure 2. Ternary diagrams. The ternary diagrams plot three element concentrations[1] (scaled and normalized to a constant sum) in bulk sediment samples from five sites in the South Pacific Gyre (colored shapes) and the compositions of average rocks, ash layers, and end-members[1, 5, 6, 19-22] (black shapes). Sites with sediment dominated by carbonate lithologies (Sites U1367 and U1368) were excluded.

Trends in the bulk sediment samples support the results and interpretations of the multivariate statistics and suggest the presence of ash and authigenic enrichment of Mg. Apatite causes the pelagic clay samples to trend toward Ca in the Ca-K-Mg plot.

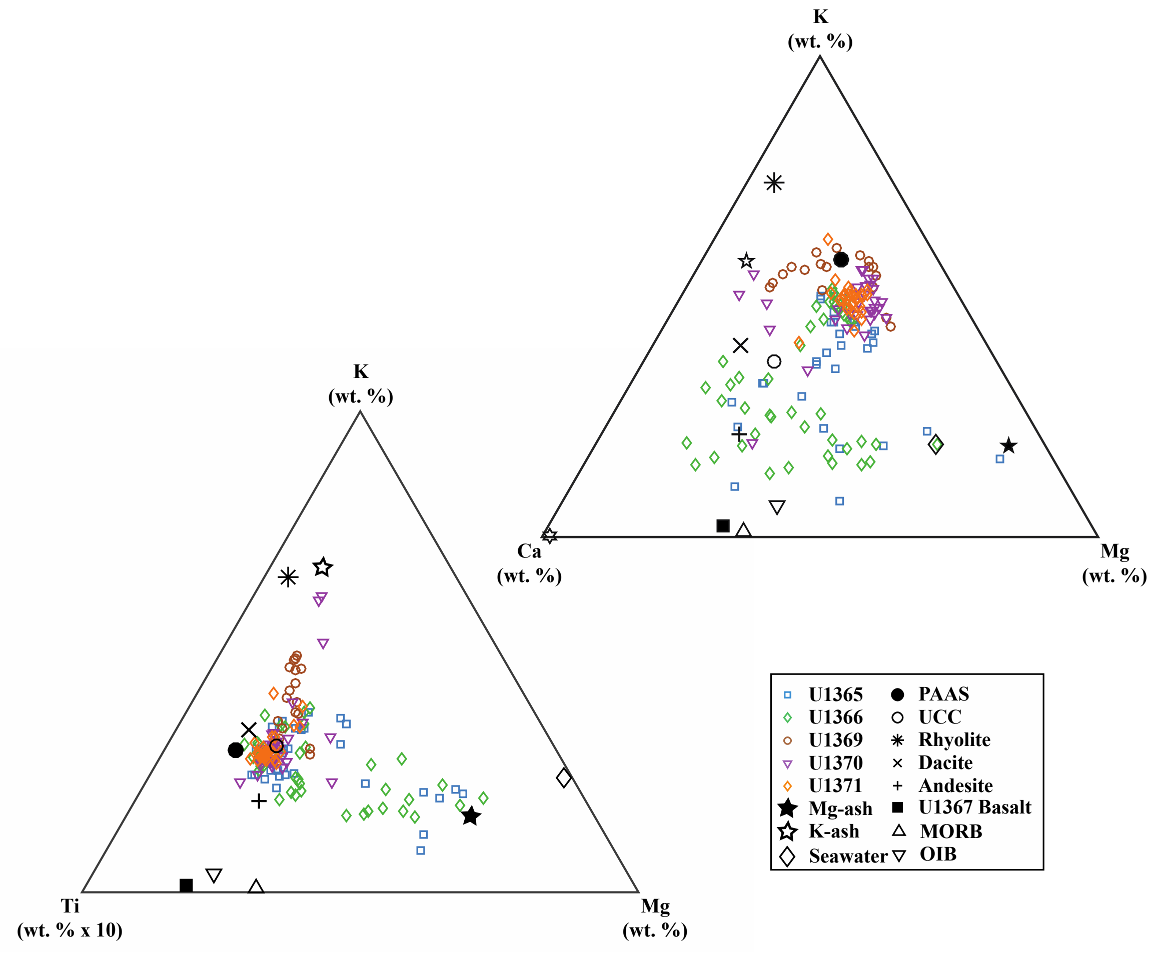

Supplementary Figure 3. CLS results at four sites in the SPG. Plotted with depth (meters below seafloor) are the modeled mass fractions of the six end-members (see legend) that comprise the deep-sea pelagic sediment from four sites (U1365, U1366, U1369, and U1370 in the panels from left to right) that were drilled during IODP Expedition 329 in the South Pacific Gyre. The constrained least squares multiple linear regression model that produced these results is described in the main and supplementary text.

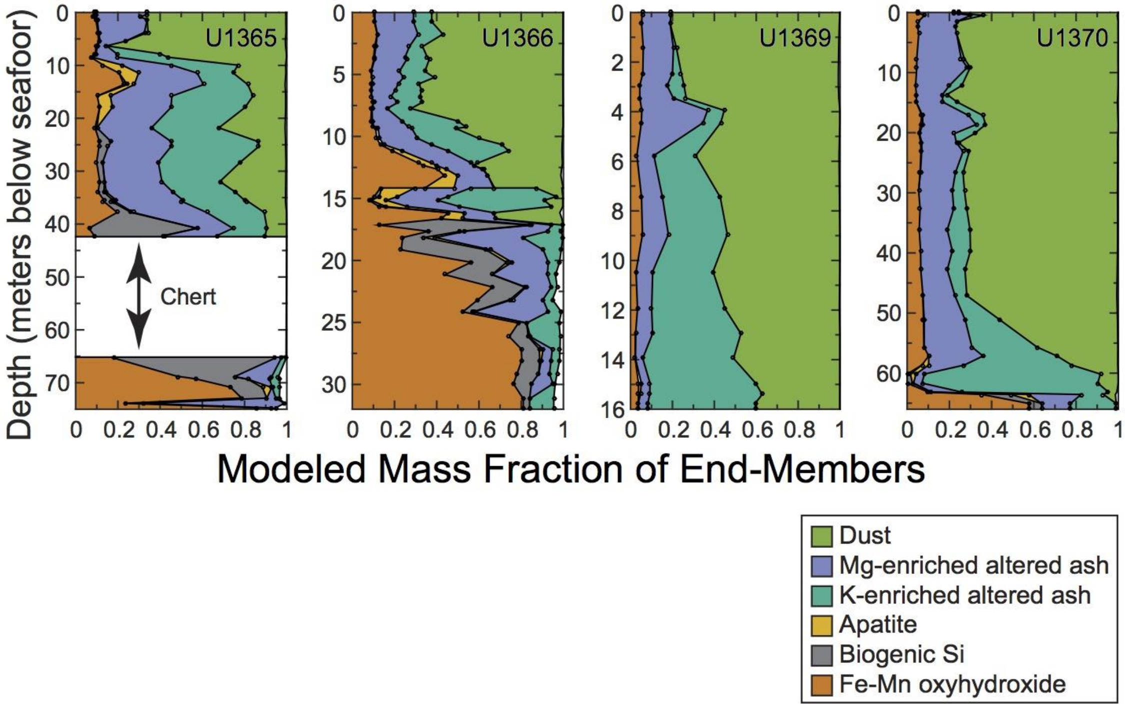

Supplementary Figure 4. Estimates of δ^{26}Mg with increasing Mg incorporation. (A.) The modeled δ^{26}Mg (‰) of an altered Mg-ash as a function of the fraction of Mg incorporated from seawater. (B.) The modeled δ^{26}Mg (‰) as a function of the total concentration of Mg (wt. %) as it is incorporated into the altered ash. The initial volcanic ash end-member is modeled as 0.1 wt. % Mg and the four series plotted use two different initial δ^{26}Mg values and two different fractionation factors ($\varepsilon^{26/24}_{s\text{-}f}$). The initial δ^{26}Mg was modeled as either bulk silicate earth (BSE, δ^{26}Mg = -0.23 ‰) or rhyolite (δ^{26}Mg = -0.46 ‰)[23]. The fractionation factor of δ^{26}Mg relative to the seawater (δ^{26}Mg = -0.82 ‰) is modeled as either $\varepsilon^{26/24}_{s\text{-}f}$ = +1.25 (ref: 24) or $\varepsilon^{26/24}_{s\text{-}f}$ = +1.34 (calculated assuming that 100% of the Mg in the Mg-ash in this study is from seawater, see discussion below).

The δ^{26}Mg value of the altered Mg-ash in this study (0.52 ‰) is heavier than would be expected if 100% of the Mg in the ash were from seawater according to the fractionation factor estimated by Higgins and Schrag (2010)[24]. Thus, we can approximate that about 100% of the Mg in the Mg-ash end-member was incorporated from seawater. These results are also supported by the bulk geochemistry of the Mg-ash. The concentration of Mg in typical rhyolite volcanic ash is very low (~0.1 wt.% Mg). The altered Mg-ash end-member in our study has 3.9 wt.% Mg (6.5 wt.% MgO) and thus 98%-100% of the Mg in the altered Mg-ash is from seawater.

In the above calculations and plots, we are assuming that the Mg in the original volcanic ash shard is retained in the authigenic phase. If the volcanic glass shard is completely recrystallized on the seafloor, 100% of the Mg in the authigenic mineral will be from

seawater and have a $\delta^{26}Mg$ of a secondary mineral forming from seawater. With this conceptual model and the other evidence that ~100% of the Mg is from seawater, we can use the $\delta^{26}Mg$ of our Mg-ash layer analysis (0.52 ‰) and state that it is fractionated +1.34 ‰ from seawater composition (-0.82 ‰). This is very close to, but slightly higher than, the fractionation predicted from porewaters (+1.25 ‰)[24].

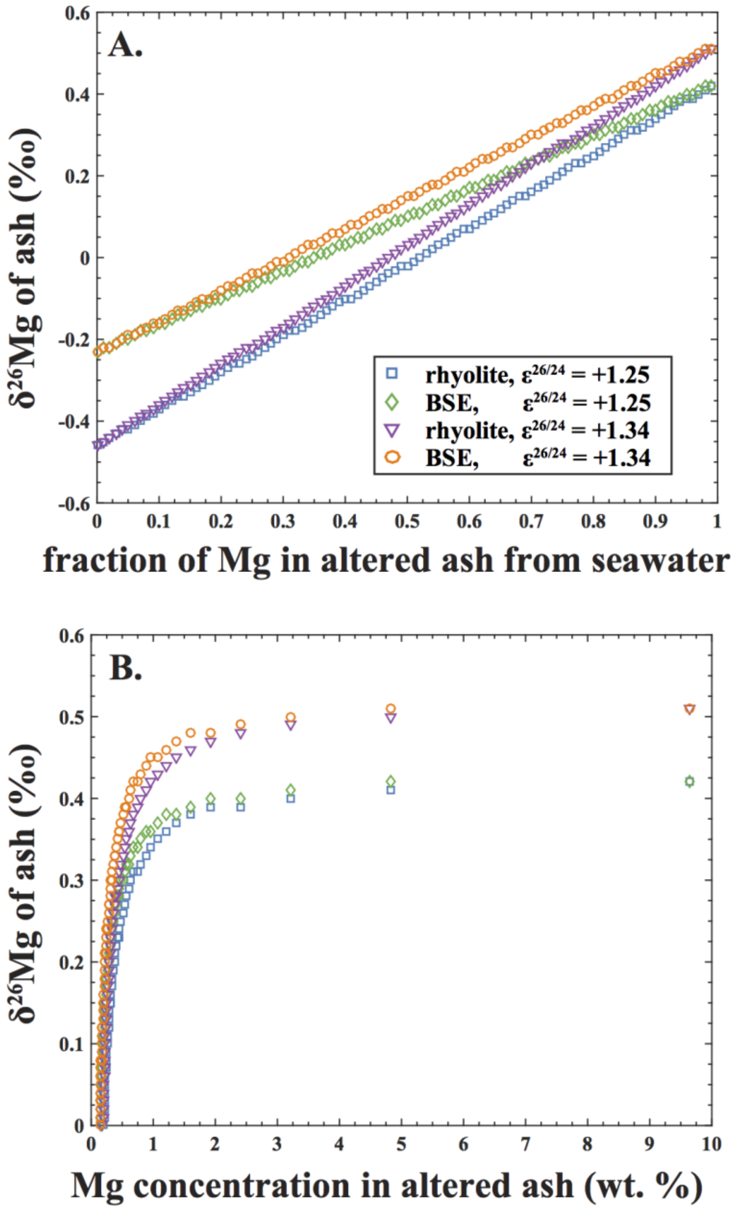

Supplementary Figure 5. Mg concentrations and δ^{26}Mg of interstitial water. Concentrations of Mg (mM)[9] and the δ^{26}Mg (‰, this study) of porewaters collected from Sites U1365, U1366, U1370, and U1371 are plotted against depth (meters below seafloor, mbsf). Both the concentrations and isotopic signatures do not vary significantly with depth indicating that Mg is not being consumed deeper in the sediment and suggesting that the authigenic clay reaction occurs on or near the seafloor.

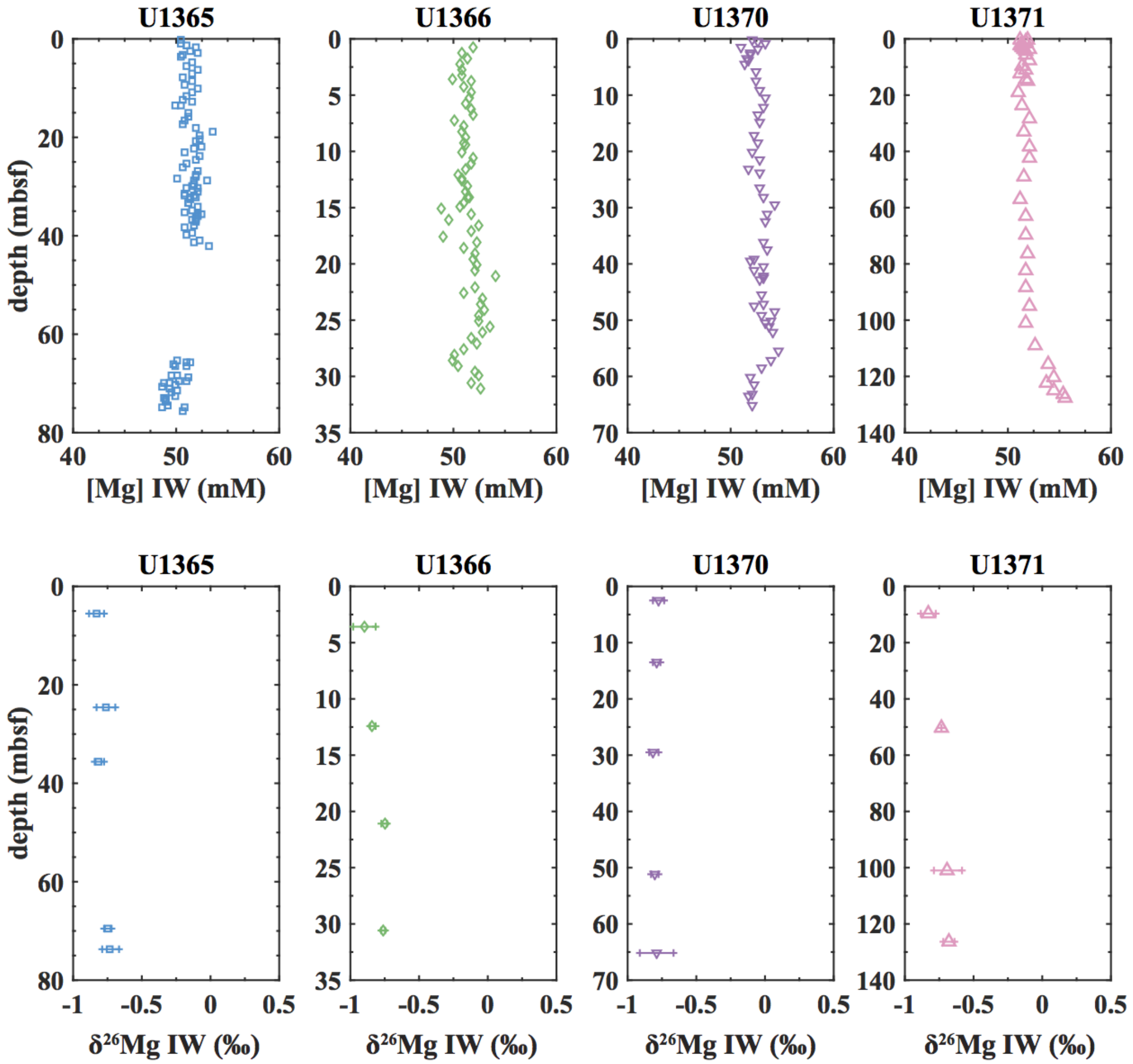

Supplementary Table 1. CLS model results and δ^{26}Mg values of samples at Site U1366. Columns 1-10: Sample identification information. Columns 11-16: Mass fractions of the six end-members used to model the bulk sediment chemistry at Site U1366. Nine major elements and Cr, Rb, and Cs are included in the element menu for the CLS analyses. Column 17: The δ^{26}Mg (‰) of each bulk sediment sample.

Exp.	Site	Hole	Core	Core Type	Sect.	Interval Top	Interval Bot	Depth	Modified Depth	Fe/Mn oxides	Apatite	Excess Si	PAAS	Mg-ash	K-ash	δ^{26}Mg
						(cm)	(cm)	(mbsf)	(mbsf)	mass fraction	mass fraction	mass fraction	mass fraction	mass fraction	mass fraction	per mil
329	U1366	D	1	H	1	30	40	0.35	0.75	0.1	0.0	0.0	0.6	0.2	0.1	-0.19
329	U1366	D	1	H	1	130	140	1.35	1.75	0.1	0.0	0.0	0.6	0.2	0.1	-0.19
329	U1366	D	1	H	2	80	90	2.35	2.75	0.1	0.0	0.0	0.7	0.2	0.1	-0.40
329	U1366	F	1	H	3	60	70	3.65	3.65	0.1	0.0	0.0	0.6	0.1	0.1	-0.21
329	U1366	D	1	H	3	30	40	3.35	3.75	0.1	0.0	0.0	0.6	0.2	0.1	-0.34
329	U1366	D	1	H	3	130	140	4.35	4.75	0.1	0.0	0.0	0.7	0.1	0.1	-0.36
329	U1366	D	1	H	4	30	40	4.85	5.25	0.1	0.0	0.0	0.6	0.1	0.1	-0.14
329	U1366	D	1	H	4	80	90	5.35	5.75	0.1	0.0	0.0	0.7	0.1	0.1	
329	U1366	D	1	H	4	130	140	5.85	6.25	0.1	0.0	0.0	0.7	0.1	0.1	
329	U1366	D	1	H	5	30	40	6.35	6.75	0.1	0.0	0.0	0.7	0.1	0.1	0.00
329	U1366	D	1	H	5	80	90	6.85	7.25	0.1	0.0	0.0	0.7	0.1	0.1	
329	U1366	D	1	H	5	130	140	7.35	7.75	0.1	0.0	0.0	0.7	0.1	0.1	-0.34
329	U1366	D	1	H	6	80	90	8.35	8.75	0.1	0.0	0.0	0.5	0.1	0.3	-0.26
329	U1366	D	1	H	7	10	20	8.85	9.25	0.1	0.0	0.0	0.5	0.2	0.3	
329	U1366	F	2	H	2	50	60	7.55	9.35	0.1	0.0	0.0	0.5	0.2	0.2	-0.24
329	U1366	D	2	H	1	30	40	9.75	10.15	0.1	0.0	0.0	0.4	0.2	0.3	-0.24
329	U1366	D	2	H	1	80	90	10.25	10.65	0.1	0.0	0.0	0.3	0.2	0.3	
329	U1366	D	2	H	1	130	140	10.75	11.15	0.2	0.0	0.0	0.3	0.2	0.3	-0.24
329	U1366	D	2	H	2	80	90	11.75	12.15	0.3	0.1	0.0	0.4	0.2	0.0	-0.19
329	U1366	F	2	H	4	50	60	10.55	12.35	0.3	0.1	0.0	0.4	0.2	0.0	-0.22
329	U1366	D	2	H	2	130	140	12.25	12.65	0.4	0.0	0.0	0.4	0.2	0.0	
329	U1366	D	2	H	3	30	40	12.75	13.15	0.4	0.1	0.0	0.3	0.1	0.0	
329	U1366	D	2	H	3	130	140	13.75	14.15	0.3	0.1	0.0	0.3	0.2	0.0	-0.16
329	U1366	F	2	H	5	80	90	12.35	14.15	0.1	0.2	0.0	0.1	0.3	0.3	0.09
329	U1366	F	2	H	6	0	10	13.05	14.85	0.1	0.1	0.0	0.0	0.2	0.5	0.19
329	U1366	D	2	H	4	80	90	14.75	15.15	0.1	0.1	0.0	0.1	0.2	0.5	0.00
329	U1366	D	2	H	4	130	140	15.25	15.65	0.1	0.1	0.0	0.1	0.2	0.4	0.07
329	U1366	F	3	H	1	30	40	14.35	16.15	0.5	0.1	0.0	0.3	0.1	0.0	-0.68
329	U1366	F	3	H	1	80	90	14.85	16.65	0.4	0.1	0.0	0.3	0.2	0.0	-0.41
329	U1366	F	3	H	1	130	140	15.35	17.15	0.1	0.0	0.7	0.0	0.1	0.1	-0.10
329	U1366	F	3	H	2	30	40	15.85	17.65	0.4	0.0	0.2	0.0	0.4	0.1	0.31
329	U1366	F	3	H	2	80	90	16.35	18.15	0.2	0.1	0.1	0.0	0.4	0.2	0.16
329	U1366	F	3	H	3	30	40	17.35	19.15	0.2	0.0	0.4	0.0	0.2	0.1	0.24
329	U1366	F	3	H	3	130	140	18.35	20.15	0.6	0.0	0.2	0.0	0.2	0.1	
329	U1366	F	3	H	4	80	90	19.35	21.15	0.4	0.0	0.2	0.0	0.3	0.0	-0.04
329	U1366	F	3	H	5	30	40	20.35	22.15	0.7	0.0	0.2	0.0	0.1	0.0	
329	U1366	F	3	H	5	130	140	21.35	23.15	0.6	0.0	0.2	0.0	0.1	0.0	
329	U1366	F	3	H	6	80	90	22.35	24.15	0.5	0.0	0.0	0.0	0.4	0.1	
329	U1366	F	3	H	7	20	30	23.25	25.05	0.8	0.0	0.0	0.0	0.0	0.2	
329	U1366	F	4	H	1	80	90	24.35	26.15	0.7	0.0	0.1	0.0	0.0	0.1	-0.41
329	U1366	F	4	H	2	30	40	25.35	27.15	0.8	0.0	0.1	0.0	0.0	0.0	
329	U1366	F	4	H	2	120	130	26.25	28.05	0.8	0.0	0.1	0.0	0.0	0.0	
329	U1366	F	4	H	3	80	90	27.35	29.15	0.8	0.0	0.1	0.0	0.1	0.0	
329	U1366	F	4	H	3	10	20	28.15	29.95	0.8	0.0	0.1	0.0	0.1	0.0	-0.32
329	U1366	F	4	H	3	40	50	29.36	31.16	0.8	0.0	0.0	0.0	0.0	0.1	

Supplementary References

1. Dunlea, A. G. *et al.* Dust, volcanic ash, and the evolution of the South Pacific Gyre through the Cenozoic. *Paleoceanography* **30,** 1078–1099 (2015).
2. Reimann, C., Filzmoser, P. & Garrett, R. Factor analysis applied to regional geochemical data: problems and possibilities. *Appl Geochem* **17,** 185–206 (2002).
3. Pisias, N. G., Murray, R. W. & Scudder, R. P. Multivariate statistical analysis and partitioning of sedimentary geochemical data sets: General principles and specific MATLAB scripts. *Geochem. Geophy. Geosy.* **5,** 4015–4020 (2013).
4. Dunlea, A. G. & Murray, R. W. Optimization of end-members used in multiple linear regression geochemical mixing models. *Geochem. Geophy. Geosy.* **16,** 4021–4027 (2015).
5. Taylor, S. R. & McLennan, S. M. *The Continental Crust: Its Composition and Evolution*. (Blackwell Scientific Publications Inc., 1985).
6. Barrett, T. J., Taylor, P. N. & Lugoqski, J. Metalliferous sediments from DSDP Leg 92: The East Pacific Rise transect. *Geochim. Cosmochim. Acta* **51,** 2241–2253 (1987).
7. Dymond, J. & Eklund, W. A microprobe study of metalliferous sediment components. *Earth Planet. Sci. Let.* **40,** 243–251 (1978).
8. Higgins, J. A. & Schrag, D. P. The Mg isotopic composition of Cenozoic seawater - evidence for a link between Mg-clays, seawater Mg/Ca, and climate. *Earth Planet. Sci. Let.* **416,** 73–81 (2015).
9. D'Hondt, S., Inagaki, F., Alvarez Zarikian, C. A.Expedition 329 Scientists. Expedition 329 Reports. *Proc. of IODP* **329,** (2011).

10. Dunlea, A. G. *et al.* Cobalt-based age models of pelagic clay in the South Pacific Gyre. *Geochem. Geophy. Geosy.* **16,** 2694–2710 (2015).

11. Dutkiewicz, A., Müller, R. D., O'Callaghan, S. & Jónasson, H. Census of seafloor sediments in the world's ocean. *Geol.* **43,** 795–798 (2015).

12. Kyte, F. T., Leinen, M., Ross Heath, G. & Zhou, L. Cenozoic sedimentation history of the central North Pacific: Inferences from the elemental geochemistry of core LL44-GPC3. *Geochim. Cosmochim. Acta* **57,** 1719–1740 (1993).

13. Renaudie, J. Quantifying the Cenozoic marine diatom deposition history: links to the C and Si cycles. *Biogeosci.* **13,** 6003–6014 (2016).

14. Berner, R. A., Lasaga, A. C. & Garrels, R. M. The carbonate-silicate geochemical cycle and its effect on atmospheric carbon dioxide over the past 100 million years. *Am. J. Sci* **283,** 641–683 (1983).

15. Kump, L. R., Brantley, S. L. & Arthur, M. A. Chemical weathering, atmospheric CO2, and climate. *Annu. Rev. Earth Planet. Sci.* **28,** 611–667 (2000).

16. Berner, R. A. Inclusion of the Weathering of Volcanic Rocks in the GEOCARBSULF Model. *Am. J. Sci.* **306,** 295–302 (2006).

17. Maher, K. & Chamberlain, C. P. Hydrologic Regulation of Chemical Weathering and the Geologic Carbon Cycle. *Science* **343,** 1502–1504 (2014).

18. Walker, J., Hays, P. B. & Kasting, J. F. A negative feedback mechanism for the long-term stabilization of Earth's surface temperature. *J Geophys Res* **86,** 9776–9782 (1981).

19. Rudnick, R. L. & Gao, S. in *Treatise on Geochemistry* (eds. Holland, H. & Turekian, K.) 1–51 (Elsevier Ltd., 2014). doi:10.1016/B978-0-08-095975-7.00301-6
20. Willbold, M. & Stracke, A. Trace element composition of mantle end-members: Implications for recycling of oceanic and upper and lower continental crust. *Geochem. Geophy. Geosy.* **7,** Q04004 (2006).
21. Gale, A., Dalton, C. A., Langmuir, C. H., Su, Y. & Schilling, J.-G. The mean composition of ocean ridge basalts. *Geochem. Geophy. Geosy.* **14,** 489–518 (2013).
22. GEOROC: Geochemistry of Rocks of the Oceans and Continents. http://georoc.mpch-mainz.gwdg.de/georoc/. Accessed March 2014.
23. Teng, F.-Z. *et al.* Interlaboratory comparison of magnesium isotopic compositions of 12 felsic to ultramafic igneous rock standards analyzed by MC-ICPMS. *Geochem. Geophy. Geosy.* **16,** 3197–3209 (2015).
24. Higgins, J. A. & Schrag, D. P. Constraining magnesium cycling in marine sediments using magnesium isotopes. *Geochim. Cosmochim. Acta* **74,** 5039–5053 (2010).
